# Epidural electrocorticography for monitoring of arousal in locked-in state

**DOI:** 10.3389/fnhum.2014.00861

**Published:** 2014-10-21

**Authors:** Suzanne Martens, Michael Bensch, Sebastian Halder, Jeremy Hill, Femke Nijboer, Ander Ramos-Murguialday, Bernhard Schoelkopf, Niels Birbaumer, Alireza Gharabaghi

**Affiliations:** ^1^Division of Functional and Restorative Neurosurgery and Division of Translational Neurosurgery, Department of Neurosurgery, Eberhard Karls University TuebingenTuebingen, Germany; ^2^Neuroprosthetics Research Group, Werner Reichardt Center for Integrative Neuroscience, Eberhard Karls University TuebingenTuebingen, Germany; ^3^Department of Empirical Inference, Max Planck Institute for Intelligent SystemsTuebingen, Germany; ^4^Department of Medical Physics, University Medical Center Utrecht, Utrecht UniversityUtrecht, Netherlands; ^5^Department of Computer Engineering, Wilhelm-Schickard Institute for Computer Science, Eberhard Karls University TuebingenTuebingen, Germany; ^6^Institute of Medical Psychology and Behavioral Neurobiology, Eberhard Karls University TuebingenTuebingen, Germany; ^7^Institute of Psychology, University of WuerzburgWuerzburg, Germany; ^8^Research Group Human Media Interaction, Department of Electrical Engineering, Mathematics and Computer Science, University of TwenteEnschede, Netherlands; ^9^Health and Quality of life Unit, Fatronik-TecnaliaSan Sebastian, Spain; ^10^Istituto di Ricovero e Cura a Carattere Scientifico, IRCCS Ospedale San CamilloVenezia, Italy

**Keywords:** electrocorticography, epidural recording, locked-in state, coma, consciousness, brain-computer interface, neuroprosthetic devices

## Abstract

Electroencephalography (EEG) often fails to assess both the level (i.e., arousal) and the content (i.e., awareness) of pathologically altered consciousness in patients without motor responsiveness. This might be related to a decline of awareness, to episodes of low arousal and disturbed sleep patterns, and/or to distorting and attenuating effects of the skull and intermediate tissue on the recorded brain signals. Novel approaches are required to overcome these limitations. We introduced epidural electrocorticography (ECoG) for monitoring of cortical physiology in a late-stage amytrophic lateral sclerosis patient in completely locked-in state (CLIS). Despite long-term application for a period of six months, no implant-related complications occurred. Recordings from the left frontal cortex were sufficient to identify three arousal states. Spectral analysis of the intrinsic oscillatory activity enabled us to extract state-dependent dominant frequencies at <4, ~7 and ~20 Hz, representing sleep-like periods, and phases of low and elevated arousal, respectively. In the absence of other biomarkers, ECoG proved to be a reliable tool for monitoring circadian rhythmicity, i.e., avoiding interference with the patient when he was sleeping and exploiting time windows of responsiveness. Moreover, the effects of interventions addressing the patient’s arousal, e.g., amantadine medication, could be evaluated objectively on the basis of physiological markers, even in the absence of behavioral parameters. Epidural ECoG constitutes a feasible trade-off between surgical risk and quality of recorded brain signals to gain information on the patient’s present level of arousal. This approach enables us to optimize the timing of interactions and medical interventions, all of which should take place when the patient is in a phase of high arousal. Furthermore, avoiding low-responsiveness periods will facilitate measures to implement alternative communication pathways involving brain-computer interfaces (BCI).

## Introduction

Assessing both the level (i.e., arousal) and the content (i.e., awareness) of pathologically altered consciousness in clinical environments is limited to evaluating patients’ motor responsiveness (Laureys et al., 2009). Neurodegenerative diseases or injuries to the central nerve system may paralyze the affected patients to such a degree that they lose any remaining ability to communicate by volitional muscle control, thereby impeding the assessment of the different dimensions of consciousness (Laureys et al., 2004).

In the case of amyotrophic lateral sclerosis (ALS), this disconnection from the environment progresses slowly. In the late stage of the disease, in which the patients are no longer able to move their body or to speak, this condition spans a transition from the locked-in state (LIS), with very limited remnants of voluntary movements such as muscle twitches or eye movements, to the completely locked-in state (CLIS), with the loss of all motor control (Kübler and Birbaumer, 2008).

This is paralleled by a decline of other physiological measures for communication, including sphincter and facial electromyography as well as oculography (Murguialday et al., 2011). Similarly, body signals mediated by the parasympathetic and sympathetic nervous system show significant abnormalities (Pinelli et al., 1995) such as decreased heart rate variation (Pisano et al., 1995), alterations of the excretory function of the salivary glands (Giess et al., 2000), disturbances of the gastrointestinal tract (Toepfer et al., 1997, 1999), and alterations of the skin responses (Masur et al., 1995).

In addition, when examining CLIS patients with electroencephalography (EEG), Kotchoubey et al. (2003) reported a large variability of event-related responses (ERP) including a complete loss of ERP. Even when provided with EEG-based brain-computer interfaces (BCIs), ALS patients are unable to retain communication once they enter CLIS. This loss of the ability to communicate might be related to the disease itself and may reflect an irreversible decline of awareness, in which case it would be unavoidable (Kübler and Birbaumer, 2008). On the other hand, such a lack of communication could also be due to methodological and technical problems that could be overcome by alternative BCI approaches (Bensch et al., 2014). Recently, a metabolic BCI based on near-infrared spectroscopy has been introduced as a promising tool for communication in CLIS (Gallegos-Ayala et al., 2014).

However, locked-in patients are known to suffer from disturbed sleep patterns with increased fragmentation during the course of the disease (Ferguson et al., 1995; Soekadar et al., 2013). These fluctuations might inherently limit the success of novel BCI approaches in the affected patients. Detecting episodes of low arousal may therefore be essential in optimizing the timing of communication attempts towards phases of higher arousal. At the same time, distorting and attenuating effects of the skull and intermediate tissue on the neural signals inherent to the classical EEG approach might be surmounted by signal detection closer to the brain (Buzsáki et al., 2012; Bensch et al., 2014). Recently, we have introduced epidural ECoG as a tool to assess attention and cognitive function in LIS (Bensch et al., 2014). However, this technique applied event-related brain-potentials at specific time points to track the long-term transition from the locked-in to the completely locked in-state, thereby not allowing a close-meshed monitoring of the patient’s current state of arousal (Bensch et al., 2014).

Thus, a novel methodology for continuous monitoring is still required to gain information on the patient’s present level of arousal and to overcome the limitations of current approaches so as to interact with patients without motor responsiveness.

## Methods

### Clinical case

The patient described in this manuscript was a well-informed 40-year-old male, late-stage ALS patient, who had been diagnosed with this disease 10 years before hospitalization and who already had a 7-year history of artificial ventilation. Being fully aware of the long-term consequences of ALS with respect to the loss of all ability to communicate in later stages of this progressive disease, he was determined to retain this ability for as long as possible. Although he was already in a late stage of this disease when he contacted us, he was still able to communicate reliably via muscle twitches. During the period of several months in which we had the opportunity to examine him on a daily basis, his status deteriorated continuously, i.e., a transition from LIS to CLIS took place. While he was initially able to gain some control of an EEG-based BCI, his BCI performance dropped to random over the course of several weeks.

Before entering this stage of his disease, the patient and his legal representative had given informed consent to implantation of an ECoG grid, both on first contact before hospitalization and during his stay in hospital before surgery. The purpose of this implantation was to monitor the patient’s arousal in the absence of other biomarkers and to develop alternative communication pathways for him with ECoG-based BCI technology. The results of the BCI communication study do not constitute part of the present report. This study was conducted in accordance with the Declaration of Helsinki and with the guidelines of the local ethics committee of the University of Tuebingen (Medical Faculty).

### Electrocorticography

**Figure 1:**
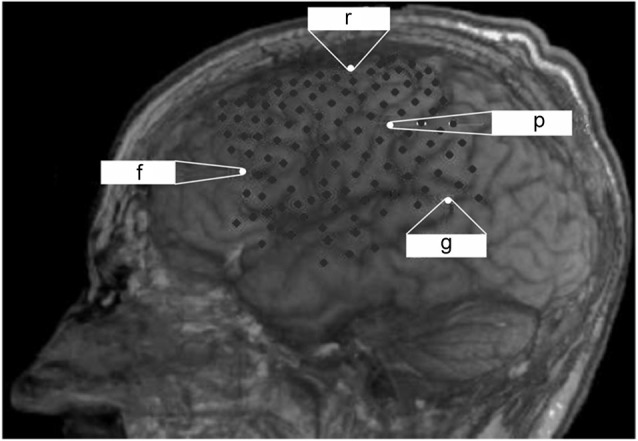
**Lateral projections of implanted ECoG recording with a grid and two electrode strips resulting in a total of 128 contacts that cover a large part of the left hemisphere**. Reference (r), ground (g) and recording channels on the frontal (f) and parietal (p) cortex for monitoring are indicated.

The observation at our institution covered a period of 6 months after ECoG grid implantation before the patient died due to a general deterioration of his medical condition unrelated to the implantation. There was no post-mortem examination, e.g., of the implant, due to the wish of the legal representative of the patient. During the observation period, the patient lost all volitional motor control for communication and entered the CLIS. For further details on this transition see Murguialday et al. (2011) and Bensch et al. (2014). Epidural ECoG recording (BrainAmp amplifier from Brain Products GmbH, Munich, Germany) was performed with a custom-made grid and two electrode strips, resulting in a total of 128 contacts (Ad-Tech Medical Instrument Corporation, Wisconsin, USA) covering parts of the left frontal, temporal and parietal cortex (Figure 1) which were externalized through subcutaneous extension wires in the ipsilateral subclavicular region. Coregistration of preoperative magnetic resonance imaging and postoperative computed tomography imaging allowed determining electrode positions with respect to the brain.

The data reported here was collected continuously over 24 h a day for 30 subsequent days. The ECoG signals were sampled at 500 Hz and data analysis (Matlab, The Mathworks™) was carried out after selecting a frontal and a parietal channel from the ECoG grid based on the signal-to-noise ratio (Figure 1). We intended to cover two different neurofunctional systems with their respective dominant frequencies (7 Hz vs. 15–17 Hz) for long-term monitoring. Moreover, previous studies have shown a significant increase in left frontal cortex glucose metabolism to correlate with improved cognitive function following amantadine medication in patients with chronic traumatic brain injury (Kraus et al., 2005). We performed a spectral analysis on the signals from these two channels, which remained constant throughout the measurements. We began by downsampling the ECoG data to 60 Hz after proper low-pass filtering to reduce the data set size. We then divided the data into 30-s epochs, estimated an autoregressive (AR) model of the order of 7 in each epoch, and derived the power spectrum from the AR coefficients in each epoch as described in Nielsen (1992). We extracted the dominant frequency over time as the frequency within the 2–30 Hz band with maximum spectral power per epoch and per channel. We then classified each 30-s epoch as a slow-wave epoch, if the dominant frequency in both channels was lower than 4 Hz and as a non-slow-wave epoch otherwise. We derived a slow-wave on/off curve by treating each consecutive epoch as a time sample and by assigning a value of 1 to slow-wave epochs and a value of 0 to non-slow-wave epochs. We then calculated the autocorrelation function of this slow-wave on/off curve to detect periodic components. We also calculated an auto-correlation function on the simultaneously recorded temperature and the heartbeat rate to detect rhythmicity.

## Results

### Clinical characteristics

Epidural ECoG was feasible and safe for monitoring cortical physiology in a late-stage ALS patient in CLIS. Despite long-term application for a period of 6 months, no implant-related complications arose; in particular, there was no infection at the site of externalization of the connection wires through the skin. Although a multi-channel device was implanted to maximize signal recording for BCI application, two recording channels (one frontal and one parietal, see Figure 1) were sufficient for longitudinal monitoring of arousal, indicating that less extended implants are feasible for this purpose in future cases.

### ECoG characteristics

**Figure 2:**
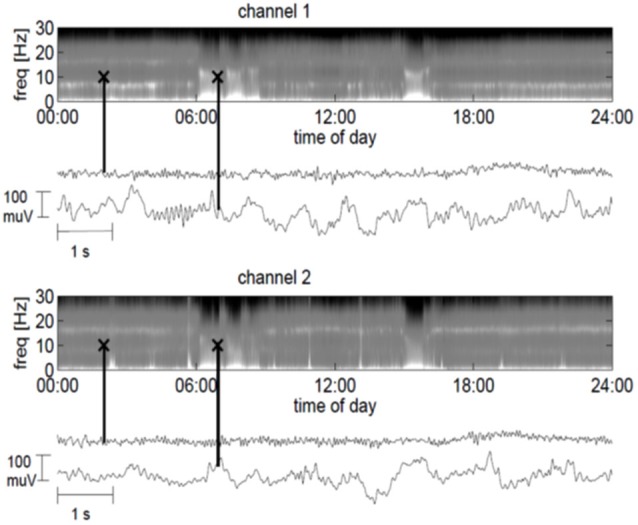
**24-hour power spectra derived from the ECoG for the frontal channel 1 (top plot) and the parietal channel 2 (lower plot), where bright colors indicate strong spectral power**. In addition, 10-s ECoG traces are indicated by black crosses in the spectrograms. The markers at 2:00 am in the spectrograms correspond to the traces directly below the spectrogram and depict the ECoG in a minimally slow state (channel 1) or normal state (channel 2). The markers at 7:00 am in the spectrograms correspond to the lower traces and show the ECoG in a slow wave period.

The frontal channel showed activity with a dominant frequency of 7 Hz, whereas the parietal channel showed an increased activity between 15 and 17 Hz. At times, the ECoG slowed down and manifested high-amplitude waves in the delta and theta range, with spindle-like activity allowing for bedside visual analysis (Figure 2). Three months after implantation we started daily amantadine medication (30 mg/day). At this time point the patient had no control over eye movements for more than 1 month, i.e., he was in the CLIS. Four days after the start of amantadine administration, the dominant frequency increased from 7 to 20 Hz (frontal channel) and from 17 to 20 Hz (parietal channel). Eleven days after the start of the amantadine administration, the patient could reliably communicate for about 30 min by means of eye movements, during which he answered negatively to the question whether he felt sorry about the surgery and replied positively to the question whether he was happy to have tried the operation. He denied wanting to die and confirmed wanting to live. After this last communication he was permanently in CLIS and was no longer able to respond to our questions.

### Arousal and circadian rhythm

**Figure 3:**
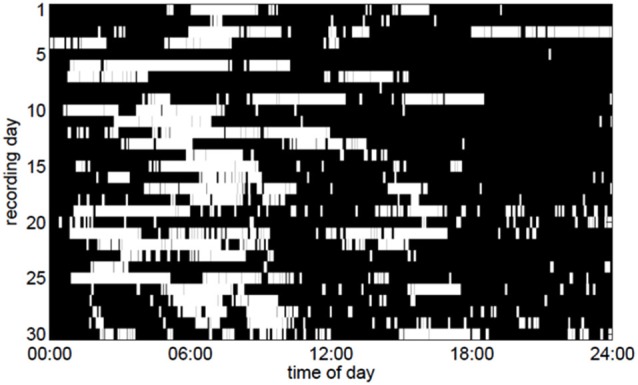
**Periods of slow-wave activity (white pixels) in the ECoG as a function of the time of day over a period of 30 days**. To make the figure clearer, only slow-wave periods lasting for at least 5 min are depicted here.

**Figure 4:**
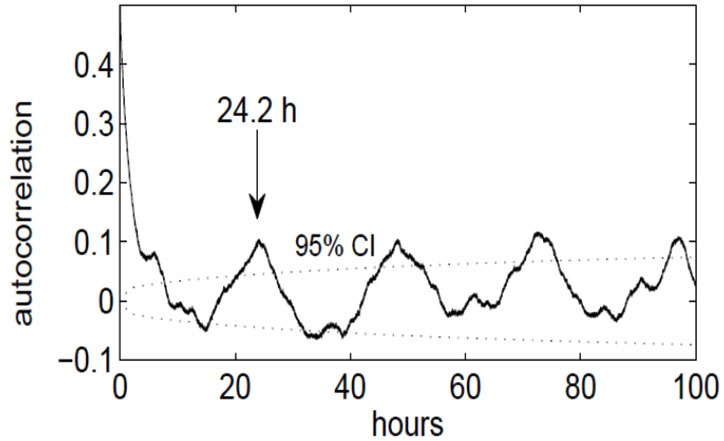
**Autocorrelation function of the slow-wave on/off curve, indicating a periodicity of approximately 24 h**. The dotted lines denote the upper and lower 95% confidence limit (*p* = 0.05), obtained by computing the effective sample size and the large-lag error by Bartlett’s formula.

We discovered that the ECoG slow-wave pattern with a frequency below 4 Hz was readily reversible by tactile stimulation, for example by repositioning the patient. This suggested that this slow-wave pattern represented phases of sleep or very low arousal similar to those in EEG recordings in healthy subjects. The slow-wave patterns were somewhat irregular. A slow wave period frequently began around midnight, although slow-wave periods were also frequently recorded during the day (Figure 3). Slow-wave periods were occasionally absent for more than 24 h. Despite these irregularities, the spectrum of the slow-wave on/off curve revealed a dominant periodicity of about 24 h (Figure 4). By contrast, neither the body temperature nor the heart rate curve revealed a circadian rhythmicity.

## Discussion

In this study, we have demonstrated that ECoG is a safe and robust tool for continuous, long-term monitoring of intrinsic oscillatory activity in a completely locked-in patient in the real-world clinical setting of an intensive care unit.

Although it is difficult to draw general conclusions from a single case, this observation supports previous findings in non-human primates demonstrating long-term stability of ECoG-based decoding of task-related oscillatory activity over several months (Chao et al., 2010). In their experiments, Chao et al. (2010) used the classical *subdural* ECoG recording technique. This technique is also applied in the majority of clinical applications, e.g., for the identification of seizure foci (McKhann et al., 2000; Kuruvilla and Flink, 2003; Luther et al., 2011) or eloquent cortex (Leuthardt et al., 2007; Miller et al., 2007, 2011). Although these *subdural* ECoG grids in patients are usually implanted for a short period of several days to 1–2 weeks only, they have been associated with significant complications. These complications increased as the number of grid electrodes increased, in particular >60 electrodes, and the longer the monitoring lasted, especially after a period of 10 days (Hamer et al., 2002). In their study, Hamer et al. (2002) also showed that infection, which occurred in 24 of 187 patients (12.2%), was the most relevant complication. These data can be intuitively explained by the fact that penetrating the dura through wires leaves a pathway for infections during the time of monitoring.

In the last few years, placing ECoG grids in the *epidural* space has been proposed to be a less invasive alternative to *subdural* grids, with most studies reporting on the decoding of task-related oscillatory activity in rodents (Uejima et al., 2009; Slutzky et al., 2010, 2011) or non-human primates (Rouse and Moran, 2009; Shimoda et al., 2012; Lee et al., 2013; Marathe and Taylor, 2013; Rouse et al., 2013; Williams et al., 2013). A few human case studies have also shown the feasibility of epidural recordings for decoding task-related oscillations during short recording sessions (Leuthardt et al., 2006; Gomez-Rodriguez et al., 2010; Gharabaghi et al., 2014a,b).

However, to date, no long-term recording of *epidural* ECoG has been reported, albeit recent experimental studies indicate signal feature detection of comparable fidelity for this modality as opposed to *subdural* ECoG (Slutzky et al., 2010; Torres Valderrama et al., 2010). Slutzky et al. (2010) used finite element modeling to compare the spatial resolution of epidurally recorded signals with those recorded from *subdural* locations. They came to the conclusion that epidural recordings yield signals of a quality similar to that of subdural recordings. Due to their reduced invasiveness, the former thus provide a more attractive source of control signals for BCIs. Moreover, Torres Valderrama et al. (2010) have reported measurements of brain signal attenuation by the human dura *in vivo* and also used signal detection theory to examine how the presence of the dura between the sources and the recording electrodes affects signal power features. It is worth mentioning that they observed no detrimental effects on signal feature detection due to the dura for noise levels typical of clinical brain signal recording equipment.

*Epidural* ECoG could therefore provide both a powerful—in terms of signal quality—and safe—in terms of surgical risk—evolution of the traditional *subdural* ECoG which itself is already known to be a good balance between the more invasive brain-penetrating approaches, i.e., intraparenchymatous micro-arrays, and the less invasive scalp EEGs (Moran, 2010; Schalk, 2010). The advantages of ECoG over EEG due to higher spatial resolution, i.e., millimeters vs. centimeters (Freeman et al., 2000; Slutzky et al., 2010), broader bandwidth, i.e., 0–500 Hz vs. 0–40 Hz (Staba et al., 2002), higher signal amplitude, i.e., 50–100 µV maximum vs. 10–20 µV, greater signal-to-noise ratio (Ball et al., 2009), and less vulnerability to artifacts such as EMG (Freeman et al., 2003), render this recording technique a powerful tool for complex clinical cases. Using simultaneous EEG/ECoG recordings in both experimental and clinical epilepsy, D’Ambrosio et al. (2009) have convincingly demonstrated that epidural and subdural ECoG signals recorded in rodents and humans, respectively, can capture short and focal epileptiform events that were undetectable by EEG despite being typically associated with subtle behavioral correlates that are easily overlooked. This renders the ECoG approach particularly suitable for ALS, where a progressive deterioration of clinical and physiological parameters, including oscillatory brain activity, takes place (Kotchoubey et al., 2003; Murguialday et al., 2011).

Following ECoG implantation in the reported patient, we made a concerted effort to analyze and apply the signals acquired with the implant. No EEG recordings were therefore available for a direct comparison between EEG and ECoG recordings. In view of the fact that the patient had a progressively deteriorating, neurodegenerative disease, a comparison of the earlier, preoperative EEG signals and the later ECoG signals after implantation was not feasible either, particularly since the patient experienced a transition from LIS to CLIS over time. Nonetheless, as we know from the literature, even mildly to moderately affected ALS patients have lower amplitudes and longer latencies of late ERP components than healthy controls (Gil et al., 1995; Münte et al., 1998; Westphal et al., 1998; Vieregge et al., 1999; Hanagasi et al., 2002; Paulus et al., 2002). For the CLIS, Kotchoubey et al. (2003) showed a large variability of the responses, including a complete loss of ERP when recording with EEG. In this context, by using ECoG recordings, we were at least able to rule out signal-distorting effects of the skull and intermediate tissue on the brain signals.

Despite long-term application of the ECoG grid for a period of 6 months—which is, to our knowledge, the longest period for human use reported in literature—no implant-related complications arose; most importantly, no infection occurred. We attributed this to the *epidural* implantation technique, and propose that this approach is more suited for monitoring chronic diseases than the subdural technique which can lead to considerably more complications the longer the device remains implanted (Hamer et al., 2002). The most obvious limitation of the present approach, however, was the necessity to externalize the connecting wires through the skin. Despite the long-term period of skin penetration, no complications related to this externalization were observed. This might be at least partly explained by the fact that we deliberately chose a region for externalization that was far enough away from the implantation site but still easily accessible for nursing, i.e., at the subclavicular region and not at the head, as is usually the case when the connecting wires of ECoG grids are externalized. Nonetheless, externalizing wires for long-term recording must be regarded as a temporary solution only. Together with industrial partners, we are therefore currently working on the development of implantable devices for clinical use which allow for wireless transmission of intracranial multichannel signals for long-term monitoring purposes. Although similar projects have already been reported (Anderson and Harrison, 2010; Guillory et al., 2011; Hirata et al., 2011; Charvet et al., 2012), no appropriate devices are currently available for clinical use.

A further limitation of the current technique was the large size of the grid and the number of electrodes implanted which necessitated a craniotomy, and hence increased invasiveness. This was based on the rationale to cover frontal (language-related), sensorimotor and temporal cortical areas for exploring different neurofunctional systems to implement BCI based communication in CLIS. Since this goal had not been realized in any other case, yet, our aim was to provide access to all relevant target structures for this purpose.

The essential precondition for any kind of BCI based communication was to identify the time periods best suited for BCI trials. This task was nontrivial since ALS patients were known to have frequent episodes of low arousal, disturbed and fragmented sleep patterns (Ferguson et al., 1995; Laureys et al., 2005; Soekadar et al., 2013) or even severe insomnia (Barthlen and Lange, 2000). Since rapid eye movement and muscle tone information was absent in the CLIS, only a coarse sleep assessment based on oscillatory recordings could be performed. Moreover, in LIS and CLIS, the operational electrophysiological definition of wakefulness and sleep was problematic because oscillations recorded might no longer have reflected the same mechanisms as in normal physiological sleep. In healthy individuals decreased arousal during sleep is accompanied by well-defined EEG changes such as slow-wave patterns (Rechtschaffen and Kales, 1968). In pathological conditions, the same slow waves do not necessarily indicate deep non-rapid eye movement or slow-wave sleep as is the case in normal sleeping individuals (Cologan et al., 2010). Sleep stage criteria in LIS and CLIS remained undefined. However, the detection of a circadian rhythmicity indicates the preservation of an—although disturbed—sleep-wake cycle, thereby allowing to differentiate the respective arousal states.

In our study, epidural recordings from one contact in the region of the dorso-lateral, left frontal cortex was sufficient to differentiate between three arousal states. Spectral analysis of the intrinsic oscillatory activity enabled us to extract state-dependent dominant frequencies at below 4 Hz, around 7 Hz and around 20 Hz, respectively. Slow wave patterns below 4 Hz commenced around midnight, although they also often occurred during the day and were unpredictable with regard to their frequency and duration. Despite these irregularities, the spectrum of the slow-wave curve revealed a dominant periodicity of about 24 h, indicating that they represented phases of sleep or very low arousal, which are common in LIS (Laureys et al., 2005). These phases were readily reversible, e.g., when the patient experienced tactile stimulation during positioning, resulting in a dominant frequency of around 7 Hz. Time periods with this dominant frequency occurred often in the afternoon and evening, and probably represented awake states. Since ECoG recordings have revealed that beta oscillations (13–30 Hz) extend far beyond primary sensorimotor regions towards the middle frontal gyrus and the pars opercularis of the frontal cortex (Groppe et al., 2013) and as TMS evoked EEG responses in the same region present a natural frequency of >20 Hz (Rosanova et al., 2009), the slowed dominant frequency (around 7 Hz) in our patient—which is often described in LISs (Patterson and Grabois, 1986)—might thus indicate decreased arousal despite the awake state in this patient and is probably related to the disease. This interpretation is supported by the increase of the dominant frequency to 20 Hz following several days of repetitive amantadine medication. This increase towards the natural frequency of the frontal cortex in our patient indicates increased arousal and is in line with earlier results in studies on the influence of amantadine in patients with chronic traumatic brain injury (Kraus et al., 2005). They showed a significant increase in left frontal cortex glucose metabolism correlated with improved cognitive function following amantadine medication. Similarly, in our patient the increase of the intrinsic oscillatory pattern was paralleled by a new episode of reliable communication by means of eye movements.

In the absence of other biomarkers, epidural ECoG proved to be a reliable tool for monitoring circadian rhythmicity in CLIS, i.e., avoiding interference with the patient when he was sleeping and exploiting time windows of responsiveness. This enabled nursing staff and therapists to adopt interventions in accordance with the patient’s individual arousal (i.e., schedule them in the afternoons and evenings). Moreover, we demonstrated that sufficient arousal monitoring could be acquired from one frontal electrode contact, indicating that future implants may require only a relatively small burr hole, thus reducing surgical risks in this vulnerable patient group even further. This methodology might also present a useful tool for monitoring the arousal in other conditions with pathologically altered consciousness whenever the patients’ motor responsiveness is compromised.

## Conclusions

We implemented a novel technique for continuous, long-term monitoring of arousal in a chronic disease without further physiological or behavioral parameters. The epidural ECoG approach presented here constitutes a feasible trade-off between surgical risk and quality of brain signals. The information provided is essential for both the social and the medical environment in dealing with expectations and planning interventions that aim to implement alternative communication pathways such as BCIs in conditions of missing motor responsiveness.

## Conflict of interest statement

The authors declare that the research was conducted in the absence of any commercial or financial relationships that could be construed as a potential conflict of interest.
